# Voluntary licensing of long‐acting HIV prevention and treatment regimens: using a proven collaboration‐ and competition‐based mechanism to rapidly expand at‐scale, sustainable, quality‐assured and affordable supplies in LMICs

**DOI:** 10.1002/jia2.26092

**Published:** 2023-07-13

**Authors:** Lobna Gaayeb, Aditi Das, Ike James, Rajesh Murthy, Sandra Nobre, Esteban Burrone, Sébastien Morin

**Affiliations:** ^1^ Medicines Patent Pool Geneva Switzerland; ^2^ Medicines Patent Pool Mumbai India

**Keywords:** affordability, generic competition, global health, long‐acting medicines, low‐ and middle‐income countries, voluntary licensing

## Abstract

**Introduction:**

Emerging long‐acting (LA) prevention and treatment medicines, technologies and regimens could be game‐changing for the HIV response, helping reach the ambitious goal of halting the epidemic by 2030. To attain this goal, the rapid expansion of at‐scale, sustainable, quality‐assured, and affordable supplies of LA HIV prevention and treatment products through accelerated and stronger competition, involving both originator and generic companies, will be essential. To do this, global health stakeholders should take advantage of voluntary licensing of intellectual property (IP) rights, such as through the United Nations‐backed, not‐for‐profit Medicines Patent Pool, as a proven mechanism to support broad access to existing HIV medicines across low‐ and middle‐income countries (LMICs).

**Discussion:**

While voluntary licensing may unlock the possibility for generic competition to take place ahead of patent expiry, there are additional elements—of amplified importance for more complex LA HIV medicines—that need to be taken into consideration. This paper discusses 10 enablers of voluntary licensing of IP rights as a model to rapidly expand at‐scale, sustainable, quality‐assured, and affordable supplies of LA HIV prevention and treatment regimens in LMICs:

1. Identifying promising LA technology platforms and drug formulations at an early developmental stage and engaging with patent holders
2. Consolidating a multidisciplinary network and strengthening early‐stage coordination and collaboration to foster innovation
3. Embedding public health considerations in product design and delivery
4. Building innovative partnerships for product development and commercialization
5. Raising awareness of and creating demand for emerging LA products
6. Estimating the market size, ensuring sufficient competition and protecting sustainability
7. Using technology transfer and hands‐on technical support to reduce product development timelines and costs
8. Exploring de‐risking mechanisms and financial incentives to support generic manufacturers
9. Optimizing strategies for generic product development and regulatory filings
10. Aligning and coordinating efforts of stakeholders across the value chain.

**Conclusions:**

Rapid access to emerging LA prevention and treatment regimens and technologies can be facilitated by voluntary licensing—catalyzed and supplemented by enabling collaborative and non‐duplicative efforts of various other stakeholders. This can effectively lead to improved—accelerated and cheaper—access to quality‐assured medicines for populations in LMICs.

## 1 INTRODUCTION

Emerging long‐acting (LA) prevention and treatment medicines, technologies and regimens could be game‐changing for the HIV response [1, 2, 3, 4, 5, 6]. The rapid expansion of at‐scale, sustainable, quality‐assured, and affordable supplies of LA treatments and technologies through accelerated and stronger competition between manufacturers will be essential. To support broad and timely access to LA medicines in low‐ and middle‐income countries (LMICs), global health stakeholders should take advantage of voluntary licensing of intellectual property (IP) rights through the United Nations‐backed, not‐for‐profit Medicines Patent Pool (MPP), as a collaboration‐ and competition‐based mechanism that has proven successful to dramatically expand access to several antiretrovirals in immediate release oral formulations in 148 countries to date [7, 8, 9, 10]. In 2020, MPP completed an exploratory phase to expand its mandate to LA technologies that could improve adherence and treatment outcomes [11, 12]. Some groups have also called on MPP to facilitate access to affordable LA medicines [13, 14, 15, 16]. Accordingly, MPP started prioritizing LA medicines, formulations and technologies for in‐licensing, and several LA technologies across the development pipeline have already been licensed [17, 18, 19, 20, 21].

## 2 DISCUSSION

### Outlining supporting elements of voluntary licensing of ip rights to unlock access to LA technologies and formulations in LMICs

Voluntary licensing of IP rights refers to “IP‐holders voluntarily granting licences to their patents or other IP,” allowing competition to take place ahead of patent expiry and supporting access to affordable medicines in LMICs [22]. Several enablers—of amplified importance for more complex LA HIV medicines and formulations—can complement and strengthen the impact of voluntary licensing [23, 24]. This paper discusses 10 enablers for products already on the market (such as cabotegravir LA, CAB‐LA, for HIV pre‐exposure prophylaxis—PrEP) and earlier‐stage LA technology platforms and drug formulations. Some of these technologies are described in the MPP Long‐Acting Therapeutics Patents and Licences Database (LAPaL [25]). Molecules with long half‐life or high potency (but no formulation feature extending their activity) are beyond this paper's scope [26, 27], while a discussion on voluntary licensing of biotherapeutics is available elsewhere [28].

### 2.1 Enabler 1: Identifying promising LA technology platforms and drug formulations at an early developmental stage and engaging with patent holders

Scoping, prioritizing and licensing promising LA technology platforms and drugs/formulations from the pipeline should take place at an early stage, well in advance of marketing authorizations, as early as phase 2 clinical trials, and in some cases at the pre‐clinical stage [27, 28, 29, 30]. There are at least four reasons for early engagement with patent holders:

1. It might be easier to negotiate access plans prior to exclusive agreements being secured between developing and commercializing entities (in particular, when developers are universities and small‐to‐medium biotechnology and pharmaceutical companies with limited commercialization capabilities). Earlier engagement with IP holders is an integral part of MPP's 2023−2025 strategy [31].
2. Early engagement can help address knowledge gaps and product design issues early [6]. It can emphasize the intended public health application(s), helping match prioritized active pharmaceutical ingredients (APIs)—such as those identified by Conference on Antiretroviral Drug Optimization (known as CADO) and Paediatric Antiretroviral Drug Optimization (known as PADO) processes—with promising LA drug delivery platforms or technologies [30].
3. As part of clinical research and product development, it may inform product specifications on needs and preferences of target populations—children and their caregivers, adolescents, pregnant and breastfeeding females, transgender individuals and people who may be on other therapies (including contraception and gender‐affirming hormone therapies)—on issues such as injection sites or potential drug−drug interactions [32, 33, 34, 35, 36, 37, 38, 39].
4. Early engagement may provide legal certainty that affordable generic versions of a product could become available in LMICs. This may be important for stakeholders in charge of downstream aspects of normative guidance, financing, procurement, rollout, uptake and scale‐up, helping ensure comprehensive rollout plans are ready (and barriers that could cause unnecessary delays are addressed) by the time generics become available [6, 40, 41].

A caveat to early engagement is the inherent risk that a product might not prove safe and/or effective. In addition, the earlier in development a product is, the less clear costs of goods, indication(s) and market size are. These are risks that, if embraced by a coordinated global partnership, may lead to substantial benefits for timely and broad access to innovative LA products in LMICs. Accordingly, strategic investments and shared risk mechanisms should be put in place to support key stakeholders leading the development lifecycle [24].

### 2.2 Enabler 2: Consolidating a multidisciplinary network and strengthening early‐stage coordination and collaboration to foster innovation

There are few approved LA products for HIV prevention (the dapivirine monthly vaginal ring and CAB‐LA) and treatment (CAB‐LA with rilpivirine LA). However, the LA HIV research and development pipeline includes several dosage forms and routes of administration, such as implants and transdermal microarray patches [5, 25, 27, 42, 43, 44]. Several collaborative platforms, tools and projects aim at supporting and complementing upstream efforts, and pipeline navigation, contributing to building a roadmap for access‐friendly development of a range of LA products of promising impact in LMICs. These initiatives bring together key contributors, including affected communities, researchers, donors, and manufacturers investing in and developing those products:

1. Research and research‐supporting initiatives, such as the Long‐Acting/Extended Release Antiretroviral Research Resource Program (LEAP) and the Centre of Excellence in Long‐Acting Therapeutics [45, 46].
2. Modelling tools, such as Teoreler, coordinated by the University of Liverpool and LEAP for pharmacokinetics modelling of LA medicines [47].
3. The dedicated Long‐Acting Technologies Community Advisory Board, convened by AfroCAB and the Treatment Action Group (TAG), supporting meaningful community engagement and ensuring clinical studies of LA technologies are conducted in ways that are safe, ethical, appropriate and responsive to community priorities and needs [48, 49].
4. A recently launched project of the Clinton Health Access Initiative (CHAI) mapping the technical feasibility and programmatic utility of HIV medicines and platform technologies pairs based on specific target product profile (TPP) attributes (P. Domanico, CHAI, personal communication)—This project is aligned with a broader initiative led by the Global Accelerator for Paediatric Formulations (GAP‐f) WHO network where GAP‐f partners will be matching priority medicines with formulation and drug delivery innovations (which may include LA technologies) to maximize their potential impact and ensure that these become scalable and affordable to LMIC settings [50].
5. LAPaL [25], coordinated by MPP, a free online resource to support innovation, API‐technology matching, and access to LA technologies and compounds that provides technical information on the development and IP status of selected LA therapeutics, as an interactive dashboard offering clinical trial and regulatory status data visualization.

### 2.3 Enabler 3: Embedding public health considerations in product design and delivery

It is critical to engage early and through the product lifecycle, with end‐user communities, caregivers and service delivery implementors (clinicians, nurses, pharmacists, community health workers and other people involved in delivering care) to include their perspectives, needs and preferences in the development of TPPs [35, 51, 52, 53, 54, 55, 56, 57, 58, 59, 60]. Product features defined by such TPPs should inform product design for compatibility with resource‐constrained settings, including to minimize the costs of starting materials as well as development, manufacturing, scale‐up, distribution, supply chain costs, and other downstream implementation and service delivery requirements and challenges.

### 2.4 Enabler 4: Building innovative partnerships for product development and commercialization

In addition to large pharmaceutical companies, engagement with originators (especially patent holders) should include smaller companies, academia and not‐for‐profit product development partnerships. Opportunities may exist for embedding broad access plans and pathways supporting scalability and economies of scale before downstream commercial agreements are in place. Sub‐licensing to manufacturers might go beyond usual generic development, as there might not yet be any originator product with marketing authorization (and none for registrational phase 3 clinical trials too), implying that a sub‐licensee may need to act as an “effective originator,” as considered in the LONGEVITY project licensing agreement [18]. Progressing voluntary licensing early might also ease obtention of freedom to operate, as LA products may involve multi‐layered patent protection: on the API, formulation (injectable, implant or other) and delivery device (as needed), in addition to multiple overlapping manufacturing process patents (e.g. on nanoparticle processes, polymers and excipients) possibly owned by several patent holders [11, 61]. Voluntary licensing terms may also support manufacturing supply chain security (e.g. for specific, sometimes proprietary, polymers and/or excipients and devices necessary to control the drug release rate) [21].

### 2.5 Enabler 5: Raising awareness of and creating demand for emerging LA products

Downstream engagement with end‐users and caregivers—including through dedicated focus groups, workshops, peer‐to‐peer communication, social media and early implementation studies—is also essential to raise awareness and sensitize potential end‐users of emerging LA products ahead of their readiness for market entry [35, 62]. This is important to ensure product acceptability and create the necessary demand (including at the government, funder and procurement agency levels) to ensure that uptake at scale will transform into public health impact. As part of raising awareness, drug product literacy is an important element, and tools such as TAG's Illustrated Glossary for Long‐Acting Technologies—as well as other community, product adoption and job aid resources (e.g. those developed to support the rollout of paediatric dolutegravir)—can be useful [63, 64].

### 2.6 Enabler 6: Estimating the market size, ensuring sufficient competition and protecting sustainability

It is also critical to carefully estimate the market size and the geographic distribution of the expected demand across countries as a function of time to assure sufficient competition (i.e. a large enough number of manufacturers) while protecting sustainability (i.e. avoiding splitting the overall market into too many non‐profitable parts). While drug price erosion correlates with the number of generic manufacturers in a given market [65], this has to be balanced with sustainability, where the maximum number of generics able to enter and stay in a given market—in a sustainable manner—depends on the overall size of that market (which can be a single market, across most or all LMICs, as in the case of pooled procurement through the Global Fund) [65, 66, 67, 68, 69]. As such, estimating short‐, mid‐ and long‐term demand forecasts (where possible), in alignment with funding and rollout plans of governments and international procurement agencies, is key, and efforts to develop projections have long been established for HIV treatment (not HIV prevention, despite progress around CAB‐LA for PrEP). Forecasting the demand and/or market size of early‐stage and/or new products with the potential to modify service delivery and/or patient acceptance paradigms may be prone to large uncertainties. Nevertheless, even scenario‐based projections can be useful in framing potential uptake and identifying the necessary levers for impact [70, 71].

### 2.7 Enabler 7: Using technology transfer and hands‐on technical support to reduce product development timelines and costs

LA products may be technologically challenging to manufacture, requiring a substantial amount of expertise and experience for successful generic version development [43, 72, 73, 74, 75, 76]. Inherent product characteristics, including complex manufacturing processes, often relying on specialized machinery or infrastructure—especially where LA properties of a formulation depend on niche formulation technologies—may also complicate product development [75, 77, 78]. Technology transfer (of any necessary process, together with documentation and professional expertise, between development and manufacturing sites) may help reduce generic product development timelines and lower their prices at launch [11]. In such cases, the transfer of documentation alone is often not enough to ensure smooth knowledge transfer and optimal process reproducibility, compared to a well‐defined plan with hands‐on technology transfer (e.g. short‐term allocation of receiving party personnel at the transferrer's site). The level of technology transfer needed depends on the know‐how that the originator may agree to provide and the experience and capability to adsorb the technology of the receiving unit. If sufficient technology transfer is not executed diligently, troubleshooting may lead to additional development work, and product quality and reproducibility, process efficiency, time to market and costs can all be at risk. Careful selection of manufacturers with specific expertise and equipment, as practiced through MPP's comprehensive Expression of Interest (EOI) blinded selection process followed by meticulous multi‐year licence management, can ensure successful and timely product development and regulatory approval [79, 80].

### 2.8 Enabler 8: Exploring de‐risking mechanisms and financial incentives to support generic manufacturers

Financial and other development incentives may help de‐risk infrastructure investments, such as for specialized equipment and sterile manufacturing lines. An example incentive programme that has worked as a complement to voluntary licensing is the Optimal project implemented by CHAI that has reduced “the time it takes for the medicines to get onto the market [by] finding ways to save on production costs and generating demand for the medicines” [81]. The paediatric dolutegravir formulation component of this project has included financial and technical support to two selected generic manufacturers (among MPP sub‐licensees), in addition to an originator‐supported technology transfer programme and regulatory work in collaboration with the US Food and Drug Administration (US FDA). This led to the fastest stringent regulatory approval of a generic HIV drug in a paediatric formulation ever [82, 83, 84].

### 2.9 Enabler 9: Optimizing strategies for generic product development and regulatory filings

Strategic development approaches (including studies establishing bioequivalence) and subsequent regulatory approval, for gatekeeping “stringent” regulatory approval (e.g. US FDA, European Medicines Agency or WHO Prequalification—PQ, as required by MPP licences and international procurement agencies) and in‐country registration, are critical [18, 19, 20, 21]. The most challenging areas for generic development of LA formulations may be establishing bioequivalence (defined by WHO as: “assurance that [the product] is clinically interchangeable with, i.e., therapeutically equivalent or bioequivalent to, the innovator product”) [85]. Challenges of bioequivalence studies for LA products include pharmacokinetic variability (i.e. differences among individuals being administered the product) resulting in large sample sizes, and long duration (stemming from a product's LA nature). These challenges have been acknowledged by regulatory bodies, that are now actively working on pilot projects to establish model‐integrated evidence approaches for bioequivalence [86]. A licence could allow generic manufacturers’ access to *in vivo* pharmacokinetic data generated by an originator, along with any *in vitro in vivo* correlation model established, thereby helping generics design their bioequivalence studies. A licence may also include originator pre‐clinical and clinical data to help generics file (and possibly adopt an abridged regulatory pathway, where it exists) in countries where the product has not yet been registered. More broadly, well‐defined regulatory pathways for generic LA products can ensure timely availability in LMICs. For this, the availability of regulatory guidelines (e.g. bioequivalence recommendations and product monographs) and prompt inclusion of prioritized products in a WHO PQ EOI can help guide generic manufacturers’ development and regulatory strategies. Challenges at the national regulatory approval level may be addressed by strengthening regulatory systems and building the capacity of regulatory assessors relative to LA products. In some cases, reliance on or recognition of stringent regulatory approval or WHO PQ may accelerate and simplify review processes in LMICs.

### 2.10 Enabler 10: Aligning and coordinating efforts of stakeholders across the value chain

Expanding at‐scale, sustainable, quality‐assured, and affordable supplies of LA HIV prevention and treatment regimens requires alignment and coordination of stakeholders’ efforts across the value chain. This includes originator pharmaceutical companies, generic manufacturers, funders, regulators, procurement and other global health agencies, access to medicines advocates, and, importantly, the communities of people living with, affected by, providing care for and/or at‐risk of HIV. To catalyze cross‐project synergies and allow the sharing of lessons learned and best practices for three early‐stage products it is supporting, Unitaid is convening the Long‐Acting Project Advisory Committee [18, 19, 21, 87]. Similarly, Unitaid, WHO, UNAIDS, Global Fund, PEPFAR and AVAC are co‐convening the Coalition to Accelerate Access to Long‐Acting PrEP [20, 71, 88]. Finally, alignment and coordination of stakeholder efforts can be aided by transparency on licensing terms (which MPP makes publicly available online) [89, 90].

## 3 CONCLUSIONS

**Figure 1:**
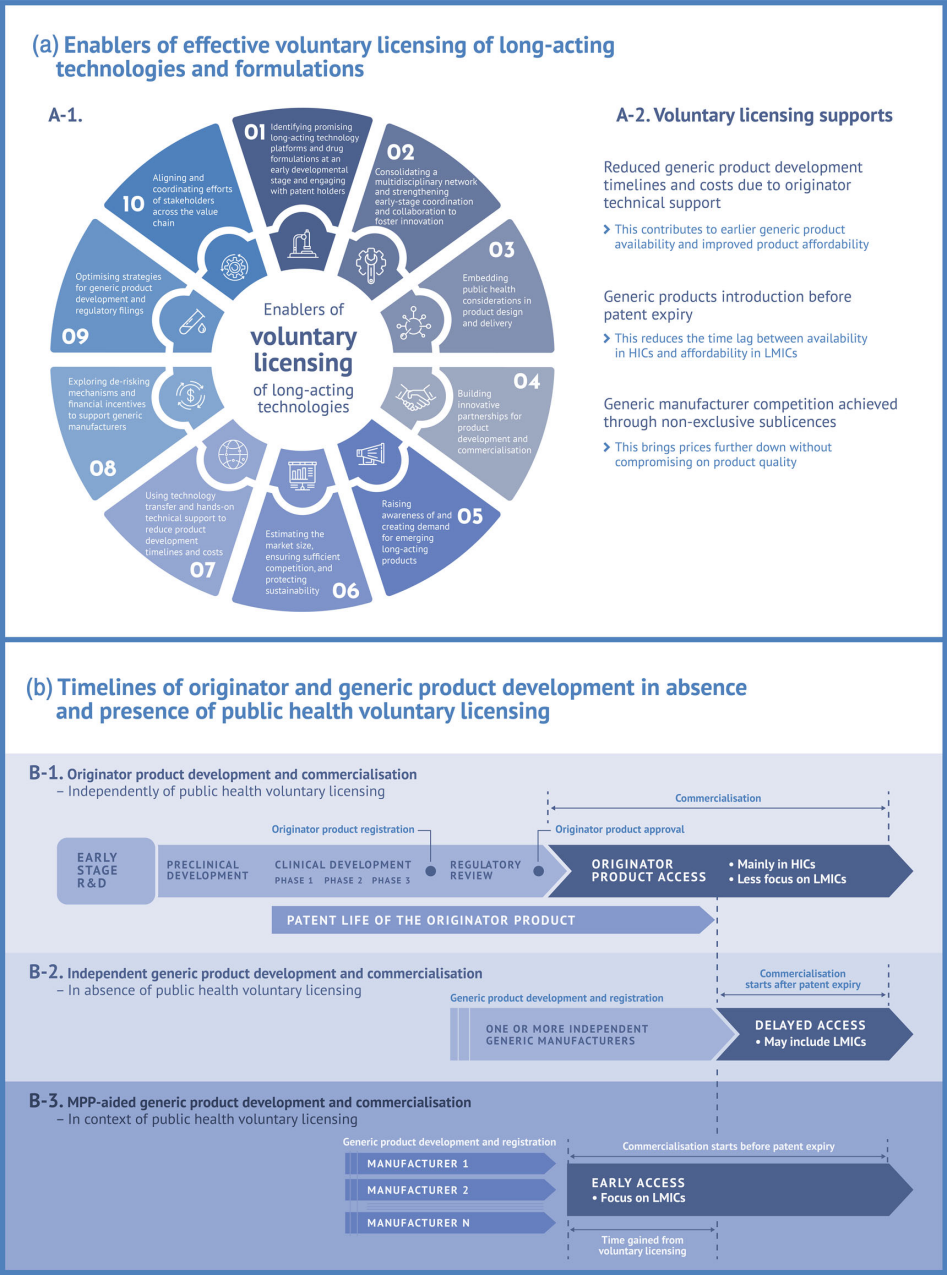
Enablers (a) and timeline benefits (b) of voluntary licensing of intellectual property rights as a model to rapidly expand at‐scale, sustainable, quality‐assured, and affordable supplies of long‐acting HIV prevention and treatment products in low‐ and middle‐income countries (LMICs). (a) Enablers of effective voluntary licensing. (A‐1) The 10 key supporting enablers discussed in the paper. (A‐2) The main beneficial effects of voluntary licensing—enabled by supporting elements in the wheel (A‐1)—that help accelerate access to more affordable products for populations in LMICs. (b) Illustrative development and commercialization timelines for originator (B‐1) and generic products: independently (following patent expiry, B‐2) and through voluntary licensing (before patent expiry, B‐3) compared in terms of the time gap between availability in the high‐income country (HIC) markets compared to affordable access to generic versions in LMIC markets. Abbreviations: R&D, research and development; MPP, Medicines patent pool.

We discussed 10 enablers of voluntary licensing that can support the rapid access to emerging LA HIV prevention and treatment regimens and technologies (Figure 1A). How these enablers, with the participation of key stakeholders, can help shorten the time between originator product availability in HICs until affordable access to quality‐assured generic versions in LMICs is illustrated in Figure 1B.

## COMPETING INTERESTS

The authors declare no competing interests.

## AUTHORS’ CONTRIBUTIONS

SM developed the paper outline; AD, IJ, LG and SM drafted the paper; LG and SM conceptualized the figures; EB, RM and SN provided strategic guidance; all authors reviewed the full paper.

## FUNDING

The authors received funding from Unitaid for this work.

## DISCLAIMER

The authors are employees of the Medicines Patent Pool.

## Data Availability

Data sharing is not applicable to this article as no datasets were generated or analysed during the current study.
